# Deep Reinforcement Learning-Based Power Allocation for Minimizing Age of Information and Energy Consumption in Multi-Input Multi-Output and Non-Orthogonal Multiple Access Internet of Things Systems

**DOI:** 10.3390/s23249687

**Published:** 2023-12-07

**Authors:** Qiong Wu, Zheng Zhang, Hongbiao Zhu, Pingyi Fan, Qiang Fan, Huiling Zhu, Jiangzhou Wang

**Affiliations:** 1 School of Internet of Things Engineering, Jiangnan University, Wuxi 214122, China; zhengzhang@stu.jiangnan.edu.cn (Z.Z.); hongbiaozhu@stu.jiangnan.edu.cn (H.Z.); 2 State Key Laboratory of Integrated Services Networks, Xidian University, Xi’an 710071, China; 3 Department of Electronic Engineering, Beijing National Research Center for Information Science and Technology, Tsinghua University, Beijing 100084, China; 4 Qualcomm, San Jose, CA 95110, USA; qf9898@gmail.com; 5 School of Engineering, University of Kent, Canterbury CT2 7NT, UK; h.zhu@kent.ac.uk (H.Z.); j.z.wang@kent.ac.uk (J.W.)

**Keywords:** deep reinforcement learning, age of information, MIMO-NOMA, Internet of Things

## Abstract

Multi-input multi-output and non-orthogonal multiple access (MIMO-NOMA) Internet-of-Things (IoT) systems can improve channel capacity and spectrum efficiency distinctly to support real-time applications. Age of information (AoI) plays a crucial role in real-time applications as it determines the timeliness of the extracted information. In MIMO-NOMA IoT systems, the base station (BS) determines the sample collection commands and allocates the transmit power for each IoT device. Each device determines whether to sample data according to the sample collection commands and adopts the allocated power to transmit the sampled data to the BS over the MIMO-NOMA channel. Afterwards, the BS employs the successive interference cancellation (SIC) technique to decode the signal of the data transmitted by each device. The sample collection commands and power allocation may affect the AoI and energy consumption of the system. Optimizing the sample collection commands and power allocation is essential for minimizing both AoI and energy consumption in MIMO-NOMA IoT systems. In this paper, we propose the optimal power allocation to achieve it based on deep reinforcement learning (DRL). Simulations have demonstrated that the optimal power allocation effectively achieves lower AoI and energy consumption compared to other algorithms. Overall, the reward is reduced by 6.44% and 11.78% compared the to GA algorithm and random algorithm, respectively.

## 1. Introduction

With the development of the Internet of Things (IoT), the base station (BS) can support the real-time applications such as disaster management, information recommendation, vehicle network, smart city, connected health and smart manufacturing by collecting the data sampled by IoT devices [1,2]. However, the amount of sampled data is enormous and the number of IoT devices is usually high; thus, the realization of these IoT applications requires a large bandwidth spectrum [3]. The multi-input multi-output and non-orthogonal multiple access (MIMO-NOMA) IoT can transmit data through the MIMO-NOMA channel to solve these problems, wherein multiple antennas are deployed at the BS to improve the channel capacity and multiple IoT devices access the common bandwidth simultaneously to improve the spectrum efficiency.

The BS collects data during discrete slots in the MIMO-NOMA IoT system. In each slot, a BS first determines the sample collection commands and allocates the transmit power for each IoT device and then sends the corresponding sample collection commands and transmission power to each IoT device. Afterwards, each IoT device determines whether to sample data from the physical world according to their sample collection commands. Then, each IoT device adopts its allocated power to transmit the sampled data to the BS over the MIMO-NOMA channel. In the transmission process, multiple IoT devices transmit the signals of the data by using the same spectrum, and therefore interference exists between different IoT devices. To eliminate the interference, the BS adopts the successive interference cancellation (SIC) technique to decode the signals from each device [4]. Specifically, the BS sorts the power of all received signals in descending order and decodes the signal with the highest received power by considering other signals as interferences. Then, the BS removes the decoded signal from the received signals and resorts the received signals to decode the next signal. The process is repeated until all signals are decoded.

The age of information (AoI) is a metric to measure the freshness of the data, which is defined as the time from the data sampling to the time when the sampled data are received [5]. In the MIMO-NOMA IoT system, the BS needs to receive data, i.e., decode the signals of the data, in a timely manner after they are sampled to provide the real-time applications; thus, a low AoI is critical in MIMO-NOMA IoT systems [6]. Furthermore, the IoT devices are energy-limited. Thus, the MIMO-NOMA IoT system should also keep its energy consumption low to prolong the working time of the IoT devices [7]. Hence, the AoI and energy consumption are two important performance metrics of the MIMO-NOMA IoT system [8]. The sample collection commands and power allocation may affect the AoI and energy consumption of the system. Specifically, for the sample collection commands, if the BS selects more IoT devices to sample, the system will consume more energy because more IoT devices consume energy to sample data. However, if the BS selects less IoT devices to sample, the data transmitted from the unselected IoT devices become obsolete, which may increase the AoI of the system. Hence, the sample collection commands affect both the AoI and energy consumption of the MIMO-NOMA IoT system. For the power allocation, if an IoT device transmits with high power, the signal transmitted by the IoT device will be decoded wherein a significant amount of signals with lower power act upon the interferences in the SIC process, which may lead to a low signal-to-interference-plus-noise ratio (SINR). Otherwise, if an IoT device transmits data with low power, the SINR may also be deteriorated due to the low transmission power. The low SINR causes a low transmission rate, which may cause a long transmission delay and a high AoI of the MIMO-NOMA IoT system. Hence, the power allocation affects the AoI of the MIMO-NOMA IoT system. Moreover, the power allocation affects the energy consumption directly. Thus, the transmission power affects both the AoI and energy consumption of the MIMO-NOMA IoT system. As mentioned above, it is critical to determine the optimal policy including sample collection commands and power allocation to minimize the AoI and energy consumption of the MIMO-NOMA IoT system. To the best of our knowledge, there is no work to minimize the AoI in the MIMO-NOMA IoT system, which motivates us to conduct this work. In the MIMO-NOMA IoT system, the allocation of transmission powers has a direct impact on the transmission rate during the SIC process. Additionally, the MIMO-NOMA channel is inherently affected by stochastic noise. Model-based algorithms struggle to construct an accurate model to describe this process, which causes the traditional model-based algorithms unsuitable to solve the problem. Deep reinforcement learning (DRL) is a type of model-free-based method that enables an agent to learn how to make sequential decisions in a complex environment to achieve a specific goal. DRL can learn the near-optimal policy by learning from the interaction between action and the environment (i.e., dynamic stochastic MIMO-NOMA IoT system) [9]. There are some existing studies on DRL-based optimization frameworks in similar systems. In [10], Zhao et al. formalized the joint optimization problem of video frame resolution selection, computation offloading and resource allocation strategy, and proposed a hierarchical reward function based on the DRL algorithm that minimizes energy consumption, maximizes quality of experience (QoE) delay and analyzes the accuracy in the IoT system. In [11], Chen et al. considered a marginalized IoT system and studied the joint caching and computing service deployment (JCCSP) problem for IoT applications driven by perceptual data. An improved method based on twin-delayed (TD) deep deterministic policy gradient (DDPG) was proposed, which achieved significant convergence performance compared to benchmarks. In general, the DRL algorithm is used to solve problems with either continuous or discrete action spaces separately. However, we focus on simplifying the joint optimization problem when the space of the sample collection commands is discrete while the space of the transmission power is continuous, to make it applicable to DDPG. We achieve this goal by establishing the relationship between sample collection commands and transmission power, and then propose a DRL-based power allocation to minimize the AoI and energy consumption of the MIMO-NOMA IoT system (The source code has been released at: https://github.com/qiongwu86/MIMO-NOMA_AoI_GA.git (7 March 2023)). The main contributions are summarized as follows:

(1) We formulated the joint optimization problem to minimize the AoI and energy consumption of the MIMO-NOMA IoT system by determining the sample selection and power allocation. Specifically, we constructed an MIMO-NOMA channel model and an AoI model to find the relationship between transmission rate and AoI of each device under the SIC mode. Additionally, we constructed an energy consumption model. Then, the joint optimization problem was formulated based on the constructed models.
(2) Then, we simplified the formulated optimization problem to make it suitable for DRL algorithms. In the formulated optimization problem, the sample selection is discrete and power allocation is continuous, which cannot be solved by the traditional DRL method and results in a challenge for optimization. We substituted the energy model and AoI model by the formulated optimization problem, merged the homogeneous terms containing sample selection and simplified the formulated problem to make it suitable to be solved by the traditional continuous-control DRL algorithm.
(3) To solve the formulated optimization problem, we first designed a DRL framework which included the state, action and reward function, and then adopted the DDPG algorithm to obtain the optimal power allocation to minimize the AoI and energy consumption of the MIMO-NOMA IoT system.
(4) Extensive simulations were carried out to demonstrate that the DDPG algorithm successfully optimizes both the AoI and energy consumption compared with other baseline algorithms.

The rest of this paper is organized as follows. Section 2 reviews the related work. Section 3 introduces the system model and formulates the optimization problem. Section 4 simplifies the formulated optimization problem and presents the near-optimal solution by DRL. We carry out some simulation to demonstrate the effectiveness of our proposed DRL method in Section 5, and conclude this paper in Section 6.

## 2. Related Work

In this section, we first review the studies about the AoI in the IoT system, and then survey the state of the arts on the MIMO-NOMA IoT system.

### 2.1. AoI in IoT

In [12], Grybosi et al. proposed the SIC-aided age-independent random access (AIRA-SIC) scheme (i.e., a slotted ALOHA fashion) for the IoT system, wherein the receiver operates SIC to reconstruct the collisions of various devices. In [13], Wang et al. focused on the problem that minimizes the weighted sum of AoI cost and energy consumption in the IoT systems by adjusting the sample policy, and proposed a distributed DRL algorithm based on the local observation of each device. In [14], Elmagid et al. aimed to minimize the AoI at the BS and the energy consumption of the generate status for the IoT devices, formulated an optimization problem based on the Markov decision process (MDP) and then proved the monotonicity property of the value function associated with the MDP. In [15], Li et al. designed a resource block (RB) allocation, modulation-selecting and coding-selecting scheme for each IoT device based on its channel condition to minimize the long-term AoI of the IoT system. In [16], Hatami et al. employed the reinforcement learning to minimize the average AoI for users in an IoT system consisting of users, energy harvesting sensors and a cache-enabled edge node. In [17], Sun et al. aimed to minimize the weighted sum of the expected average AoI of all IoT devices, propulsion energy of unmanned aerial vehicle (UAV) and transmission energy of IoT devices by determining the UAV flight speed, UAV placement and channel resource allocation in the UAV-assisted IoT system. In [18], Hu et al. considered an IoT system wherein the UAVs take off from a data center to deliver energy and collect data from sensor nodes, and then fly back to the data center. They minimized the AoI of the collected data by dynamic programming (DP) and ant colony (AC) heuristic algorithms. In [19], Emara et al. developed a spatio-temporal framework to evaluate the peak AoI (PAoI) of the IoT system, and compared the PAoI under the time-triggered traffic with event-triggered traffic. In [20], Lyu et al. considered a marine IoT scenario, wherein the AoI is utilized to represent the impact of the packet loss and transmission delay. They investigated the relationship between AoI and state estimation error, and minimized the state estimation error by the decomposition method. In [21], Wang et al. investigated the impact of AoI on the system cost which consists of control cost and communication energy consumption of the industrial-Internet-of-Things (IIoT) system. They proved that the upper bound of cost is affected by the AoI. In [22], Hao et al. maximized the sum of the energy efficiency of the IoT devices under the constraints of AoI by optimizing the transmission power and channel allocation in a cognitive radio-based IoT system. However, none of these works have taken the MIMO-NOMA channel into account.

### 2.2. MIMO-NOMA IoT System

In [23], Yilmaz et al. proposed a user selection algorithm for the MIMO-NOMA IoT system to improve the sum data rate, and adopted the physical layer network coding (PNC) to improve the spectral efficiency. In [24], Shi et al. considered the downlink of the MIMO-NOMA IoT networks and studied the outage probability and goodput of the system with the Kronecker model. In [25], Wang et al. proposed that the resource allocation problem consists of the optimal beamforming strategy and power allocation in the MIMO-NOMA IoT system, wherein the beamforming optimization is solved by the zero-forcing method, and after that the power allocation is solved by the convex functions. In [26], Han et al. proposed a novel millimeter wave (mmWave) positioning MIMO-NOMA IoT system and proposed the position error bound (PEB) as a novel performance evaluation metric. In [27], Zhang et al. considered the massive MIMO and NOMA to study the performance of the IoT system, and calculated the closed-form function for spectral and energy efficiencies. In [28], Chinnadurai et al. considered the heterogeneous cellular network and formulated a problem to maximize the energy efficiency of the MIMO-NOMA IoT system, wherein the non-convex problem was solved based on the branch and reduced-bound (BRB) approach. In [29], Gao et al. considered the mmWave massive MIMO and NOMA IoT system to maximize the weighted sum transmission rate by optimizing the power allocation, and then solved the problem by the convex method. In [30], Feng et al. considered an UAV-aided MIMO-NOMA IoT system and regarded an UAV as the BS. They formulated the problem to maximize the sum transmission rate of the downlink by optimizing the placement of UAVs, beam pattern and transmission power, and then solved the problem by convex methods. In [31], Ding et al. designed a novel MIMO-NOMA system consisting of two different users, wherein user one should be served with strict quality-of-service (QoS) requirement, and user two accesses the channel by the non-orthogonal way opportunistically; thus, the requirement that small packets of user one in the IoT system should be transmitted in time can be met. In [32], Bulut et al. proposed the water cycle algorithm (WCA) based on the energy allocation method for MIMO-NOMA IoT systems. Their simulation results demonstrated that the proposed method performs better than empirical search algorithm (ESA) and genetic algorithm (GA). In [33], Ullah et al. proposed a power allocation algorithm based on DDPG to maximize energy efficiency in MIMO-NOMA next-generation Internet-of-Things (NG-IoT) networks. Their simulation results demonstrated that the proposed method achieved better performance compared with random algorithms and greedy algorithms. However, these works have not considered the AoI of the MIMO-NOMA IoT system.

As mentioned above, there is no work considering the joint optimization problem of age of information and energy in the MIMO-NOMA IoT system, which motivates us to conduct this work. The comparison of the related works is shown in Table 1.

## 3. System Model and Problem Formulation

### 3.1. Scenario Description

**Figure 1:**
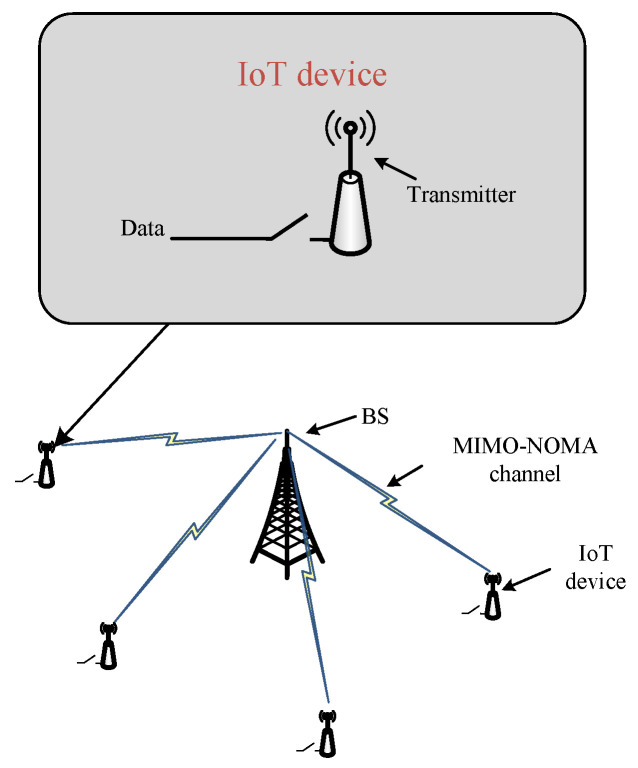
MIMO-NOMA IoT system.

The network scenario is illustrated in Figure 1. We consider a MIMO-NOMA IoT system consisting of a BS with *K* antennas and a set M={1,⋯,m,⋯,M} of the single-antenna IoT devices. Here, each IoT device is embedded with a sensor and a transmitter. The time duration is divided into *T* slots, each of which is τ. The set of slots is denoted as T={1,⋯,t,⋯,T}. At the beginning of each slot *t*, the BS determines the policy (including the sample collection commands of each device *m*, denoted as $s_{m,t}$, and the transmission power of each device *m*, denoted as $p_{m,t}$) and then sends $s_{m,t}$ and $p_{m,t}$ to each device *m*. If $s_{m,t}=1$, device *m* will sample data in slot *t*, and transmit the data to the BS with transmission power $p_{m,t}$ over the MIMO-NOMA channel. This action reduces the AoI, but also incurs a cost in terms of energy consumption. Otherwise, it does not sample data in slot t; therefore, it does not consume energy for sampling and transmission, while increasing the AoI due to a lack of updates. The key notations are listed in Table 2. Next, we will construct the MIMO-NOMA channel model.

### 3.2. MIMO-NOMA Channel Model

Let $c_{m,t}$ be the data symbol of device *m* in slot *t* with 1 as variance; thus, the signal of the data transmitted by device *m* is $\sqrt{p_{m,t}}c_{m,t}$. Let $h_{m}\left(t\right)\in C^{K\times 1}$ be the channel power gain between the BS and device *m* in slot *t*; thus, the corresponding signal received by the BS is $h_{m}\left(t\right)\sqrt{p_{m,t}}c_{m,t}$. Note that $c_{m,t}$ is unknown for the BS, so that it is difficult for the BS to calculate the received signal. Hence, the BS needs to adopt the SIC technology to decode the received signal transmitted by each device, which is expressed as

(1) $$\begin{array}{c} y\left(t\right)=\sum_{m\in M}h_{m}\left(t\right)\sqrt{p_{m,t}}c_{m,t}+n\left(t\right)\\ p_{m,t}\in \left[0,P_{m,max}\right],\forall m\in M,\forall t\in T \end{array}\;,$$

where $n\left(t\right)\in C^{K\times 1}$ is the complex additive white Gaussian noise (AWGN) with variance $\sigma_{R}^{2}$ and $P_{m,max}$ is the maximum transmission power of device *m*.

In [34,35], the authors adopted $h_{m}\left(t\right)$ estimated by the deep neural network or minimum mean square error method in the SIC process and demonstrated its efficiency. In addition, the BS also knows $p_{m,t}$; thus, the BS can calculate the power of the received signal transmitted by device *m* as

(2) $$\Gamma_{m,t}=p_{m,t}\left|\right|h_{m}\left(t\right){\left|\right|}^{2}.$$

Then, the BS decodes the received signal transmitted by each device sequentially. For one iteration, the BS decodes the signal with the highest received power from y(t) while considering the other signals as interference, and then removes the decoded signal from y(t) and starts the next iteration until all signals are decoded.

For instance, in an iteration, the received power of the signal transmitted by device *m* is the highest among the signals without being decoded. Denote $I_{m}=\left\{k\in M|\Gamma_{k,t}<\Gamma_{m,t}\right\}$ as the set of devices whose signals’ received powers is less than device *m*. Thus, the signal transmitted by each device $k\in I_{m}$ is deemed as the interference. In this case, y(t) is rewritten as

(3) $$\begin{array}{c} y\left(t\right)=h_{m}\left(t\right)\sqrt{p_{m,t}}c_{m,t}+\sum_{k\in I_{m}}h_{k}\left(t\right)\sqrt{p_{k,t}}c_{k,t}+n\left(t\right), \end{array}$$

where $\sum_{k\in I_{m}}h_{k}\left(t\right)\sqrt{p_{k,t}}c_{k,t}$ indicates the interference; thus, the signal-to-interference-plus-noise ratio (SINR) of device *m* is calculated as

(4) $$\begin{array}{cc} \gamma_{m,t}\; & =\frac{p_{m,t}\left|\right|h_{m}\left(t\right){\left|\right|}^{2}}{\sum_{k\in I_{m}}p_{k,t}\left|\right|h_{k}\left(t\right){\left|\right|}^{2}+\sigma_{R}^{2}}\\ \; & =\frac{\Gamma_{m,t}}{\sum_{k\in I_{m}}\Gamma_{k,t}+\sigma_{R}^{2}} \end{array}\;.$$

The transmission rate of device *m* in slot *t* can be derived according to Shannon capacity formula, i.e.,

(5) $$\pi_{m,t}=W\log_{2}\left(1+\gamma_{m,t}\right),$$

where *W* is the bandwidth of the MIMO-NOMA channel.

### 3.3. AoI Model

Denote $\phi_{m,t}$ as the AoI at device *m* in slot *t*, which can be calculated as

(6) $$\phi_{m,t}=\left\{\begin{array}{c} 0,\;\;\;s_{m,t}=1\\ \phi_{m,t-1}+\tau ,\;otherwise \end{array}.\right.$$

According to Equation (6), at the beginning of slot *t*, if device *m* samples data, i.e., $s_{m,t}=1$, $\phi_{m,t}$ will be reset to 0. Otherwise, $\phi_{m,t}$ will be increased by τ.

Device *m* will transmit data with transmission power $p_{m,t}$ after sampling data. If the data volume transmitted within a slot is larger than the packet size *Q*, i.e., $\pi_{m,t}\cdot \tau \geq Q$, device *m* will transmit the data successfully; otherwise, the transmission fails. Denoting $u_{m,t}=1$ as a successful transmission by device *m* in slot *t* and $u_{m,t}=0$ as an unsuccessful transmission, we have

(7) $$u_{m,t}=\left\{\begin{array}{c} 1,\;\pi_{m,t}\cdot \tau \geq Q\\ 0,\;otherwise \end{array}.\right.$$

According to [36], if a transmission from device m is successful, the AoI at the BS equals the aggregation of AoI at device m and the transmission delay. Otherwise, the AoI at the BS is increased by a slot; therefore, we have

(8) $$\Phi_{m,t}=\left\{\begin{array}{c} \phi_{m,t}+l_{m,t},\;u_{m,t}=1\\ \Phi_{m,t-1}+\tau ,\;otherwise \end{array},\right.$$

where $l_{m,t}$ is the transmission delay of device *m* in slot *t*, which is calculated as

(9) $$l_{m,t}=\frac{Q}{\pi_{m,t}}.$$

The AoI of the MIMO-NOMA IoT system is measured by averaging the AoI of all devices at the BS, i.e.,

(10) $$\bar{\Phi }=\frac{1}{T}\sum_{t\in T}\sum_{m\in M}\Phi_{m,t}.$$

### 3.4. Energy Consumption Model

Since each device consumes energy in data sampling and transmission, the energy consumption of device *m* in slot *t* can be calculated as

(11) $$\varepsilon_{m,t}=s_{m,t}C_{s}+p_{m,t}l_{m,t},$$

where $C_{s}$ is the energy consumption for data sampling [13], and $p_{m,t}l_{m,t}$ is the energy consumption for transmission.

The BS has a stable power supply; hence, the energy consumption of the BS is sufficient and thus it is not taken into account in the system. Hence, the energy consumption of the MIMO-NOMA IoT system is measured by averaging the energy consumption of all devices, i.e.,

(12) $$\bar{\varepsilon }=\frac{1}{T}\sum_{t\in T}\sum_{m\in M}\varepsilon_{m,t}.$$

### 3.5. Problem Formulation

In this work, our target is to minimize the AoI and energy consumption of the MIMO-NOMA IoT system, which is impacted by $p_{m,t}$ and $s_{m,t}$. Therefore, the optimization problem is formulated as

(13) $$\begin{array}{cc} \; & \min_{s_{t},p_{t}}\left[\gamma_{a}\bar{\Phi }+\gamma_{e}\bar{\varepsilon }\right] \end{array}$$

(13a) $$\begin{array}{cc} s.t.\; & p_{m,t}\in \left[0,P_{m,max}\right],\forall m\in M,\forall t\in T, \end{array}$$

(13b) $$\begin{array}{cc} \; & s_{m,t}\in \left\{0,1\right\},\forall m\in M,\forall t\in T, \end{array}$$

where $s_{t}=\left\{s_{1,t},\cdots ,s_{m,t},\cdots ,s_{M,t}\right\}$ and $p_{t}=\left\{p_{1,t},\cdots ,p_{m,t},\cdots ,p_{M,t}\right\}$, $\gamma_{a}$ and $\gamma_{e}$ are the non-negative weighted factors. Next, we will present a solution to the problem based on DRL.

## 4. DRL Method for Optimization of Power Allocation

In this section, we solve the optimization problem based on the DRL. First, we design the DRL framework including the state, action and reward function, wherein the relationship between the sample collection commands and transmission power is derived to facilitate the DRL algorithm in solving the problem. Then, we obtain a near-optimal power allocation based on the DRL algorithm.

### 4.1. DRL Framework

The DRL framework consists of three significant elements: state, action and reward function. For each slot, the agent observes the current state and takes the current action according to policy μ, where policy μ yields the action based on the state. Then, the agent calculates its corresponding reward under the current state and action according to the reward function, while the current state in the environment transits to the next state. Next, we will design agent, state action and reward function based on DRL [37], respectively.

- **Agent:** In each slot, the BS determines the transmission power and sample collection commands of each device based on its observation; thus, we consider the BS as the agent.
- **State:** In the system model, the state $o_{t}$ observed by the BS in slot *t* is defined as
  (14) $$o_{t}=\left[o_{1,t},\cdots ,o_{m,t},\cdots ,o_{M,t}\right],$$
  where $o_{m,t}$ represents the observation of device *m*, which is designed as
  (15) $$o_{m,t}=\left[u_{m,t-1},\gamma_{m,t-1},\Phi_{m,t-1}\right].$$
  Here, $u_{m,t-1}$, $\gamma_{m,t-1}$ and $\Phi_{m,t-1}$ can be calculated by the BS from the historical data in slot t−1.
- **Action:** According to the problem formulated in Equation (13), the action in slot *t* is set as
  (16) $$a_{t}=\left[s_{t},p_{t}\right].$$
  The two traditional DRL algorithms, namely DDPG and Deep Q-Learning (DQN), are suitable for continuous and discrete action space, respectively. However, $s_{m,t}\in \left\{0,1\right\}$ and $p_{m,t}\in \left[0,P_{m,max}\right]$ in Equation (16); thus, the space of $s_{t}$ is discrete while the space of $p_{t}$ is continuous. Hence, the optimization problem can neither be solved by DQN nor DDPG. Next, we will investigate the relationship between $p_{m,t}$ and $s_{m,t}$ to handle this dilemma.
  Substituting Equations (10) and (12) by Equation (13), the optimization objective is rewritten as Equation (17a). Then, substituting Equations (8) and (11) by Equation (17a), we can obtain Equation (17b), where $\phi_{m,t}$ is denoted as $\phi_{m,t}\left(s_{m,t}\right)$ to indicate that it is the function of $s_{m,t}$. Then, by reorganizing Equation (17b), we have Equation (17c). The first term of Equation (17c) is related with $s_{m,t}$; next, we rewrite the first term of Equation (17c) as Equation (18) to investigate the relationship between $s_{m,t}$ and $p_{m,t}$. Substituting Equation (6) by Equation (18), we have Equation (18a). Then, by merging the homogeneous terms containing $s_{m,t}$ and $\gamma_{a}$ in Equation (18a), respectively, we have Equation (18b). Let $C_{m,t,1}=\gamma_{e}C_{s}-\gamma_{a}u_{m,t}\left(\phi_{m,t-1}+\tau \right)$ and $C_{m,t,2}=\gamma_{a}\left[u_{m,t}\left(\phi_{m,t-1}+\tau \right)+\left(1-u_{m,t}\right)\left(\Phi_{m,t-1}+\tau \right)+u_{m,t}l_{m,t}\right]+\gamma_{e}p_{m,t}l_{m,t}$; thus, Equation (18b) is rewritten as Equation (18c), where $C_{m,t,1}$ is the coefficient for homogeneous terms containing $s_{m,t}$ in Equation (18b), and $C_{m,t,2}$ contains all terms without $s_{m,t}$ in Equation (18b).
  (17) $$\begin{array}{cc} \; & \gamma_{a}\bar{\Phi }+\gamma_{e}\bar{\varepsilon } \end{array}$$
  (17a) $$\begin{array}{cc} = & \frac{1}{T}\sum_{t\in T}\sum_{m\in M}\left[\gamma_{a}\Phi_{m,t}+\gamma_{e}\varepsilon_{m,t}\right] \end{array}$$
  (17b) $$\begin{array}{cc} = & \frac{1}{T}\sum_{t\in T}\sum_{m\in M}\left[\gamma_{a}\left[\left(1-u_{m,t}\right)\left(\Phi_{m,t-1}+\tau \right)+u_{m,t}\left(\phi_{m,t}\left(s_{m,t}\right)+l_{m,t}\right)\right]+\gamma_{e}\left(s_{m,t}C_{s}+p_{m,t}l_{m,t}\right)\right] \end{array}$$
  (17c) $$\begin{array}{cc} = & \frac{1}{T}\sum_{t\in T}\sum_{m\in M}\left[\left[\gamma_{a}u_{m,t}\phi_{m,t}\left(s_{m,t}\right)+\gamma_{e}s_{m,t}C_{s}\right]+\gamma_{a}\left[\left(1-u_{m,t}\right)\left(\Phi_{m,t-1}+\tau \right)+u_{m,t}l_{m,t}\right]+\gamma_{e}p_{m,t}l_{m,t}\right] \end{array}$$
  (18) $$\begin{array}{cc} \; & \gamma_{a}\Phi_{m,t}\left(s_{m,t},p_{m,t}\right)+\gamma_{e}\varepsilon_{m,t}\left(s_{m,t},p_{m,t}\right) \end{array}$$
  (18a) $$\begin{array}{cc} = & \gamma_{a}u_{m,t}\left(1-s_{m,t}\right)\left(\phi_{m,t-1}+\tau \right)+\gamma_{e}s_{m,t}C_{s}+\gamma_{a}\left[\left(1-u_{m,t}\right)\left(\Phi_{m,t-1}+\tau \right)+u_{m,t}l_{m,t}\right]+\gamma_{e}p_{m,t}l_{m,t} \end{array}$$
  (18b) $$\begin{array}{cc} = & s_{m,t}\left[\gamma_{e}C_{s}-\gamma_{a}u_{m,t}\left(\phi_{m,t-1}+\tau \right)\right]+\gamma_{a}\left[u_{m,t}\left(\phi_{m,t-1}+\tau \right)+\left(1-u_{m,t}\right)\left(\Phi_{m,t-1}+\tau \right)+u_{m,t}l_{m,t}\right]+\gamma_{e}p_{m,t}l_{m,t} \end{array}$$
  (18c) $$\begin{array}{cc} = & s_{m,t}C_{m,t,1}+C_{m,t,2} \end{array}$$
  In $C_{m,t,1}$ and $C_{m,t,2}$, $\phi_{m,t-1}$ can be calculated by the BS based on the historical data in slot t−1 [13] and $\Phi_{m,t-1}$ is known for the BS. In addition, the BS can calculate $\gamma_{m,t}$ according to Equations (4) and (5); thus, $u_{m,t}$ and $l_{m,t}$ can be further calculated according to Equations (7) and (9) given $p_{m,t}$, which means that $C_{m,t,1}$ and $C_{m,t,2}$ depend on $p_{m,t}$ and are independent of $s_{m,t}$. Hence, the optimal sample collection commands to minimize $s_{m,t}C_{m,t,1}+C_{m,t,2}$, denoted as $s_{m,t}^{*}$, are achieved when the term $s_{m,t}C_{m,t,1}$ is at its minimum; thus, we have
  (19) $$s_{m,t}^{*}=\left\{\begin{array}{c} 1,\;C_{m,t,1}<0\\ 0,\;otherwise \end{array}.\right.$$
  Hence, the optimal sample collection commands can be determined according to Equation (19) when $p_{m,t}$ is given and Equation (13) can be rewritten as
  (20) $$\begin{array}{cc} \; & \min_{p_{t}}\left[\gamma_{a}\bar{\Phi }+\gamma_{e}\bar{\varepsilon }\right] \end{array}$$
  (20a) $$\begin{array}{cc} s.t.\; & p_{m,t}\in \left[0,P_{m,max}\right],\forall m\in M,\forall t\in T, \end{array}$$
  (20b) $$\begin{array}{cc} \; & s_{m,t}^{*}=\left\{\begin{array}{c} 1,\;C_{m,t,1}<0\\ 0,\;otherwise \end{array}.\right. \end{array}$$
  According to Equation (20), the action $a_{t}$ is only reflected by $p_{t}$. Therefore, DDPG, which is suitable for the continuous action space, can be employed as the desired algorithm to solve the optimization problem in Equation (20).
- **Reward function:** The BS aims to minimize the AoI and energy consumption of the MIMO-NOMA IoT system, and the target of the DDPG algorithm is to maximize the reward function. Therefore, the reward function in slot *t* can be defined as
  (21) $$r_{t}\left(o_{t},p_{t}\right)=-\sum_{m\in M}\left[\gamma_{a}\Phi_{m,t}+\gamma_{e}\varepsilon_{m,t}\right].$$
  Furthermore, the expected long-term discounted reward of the system can be defined as
  (22) $$J\left(\mu \right)=E\left[\sum_{t=1}^{T}\beta^{t-1}r_{t}\left(o_{t},p_{t}\right){|}_{p_{t}=\mu \left(o_{t}\right)}\right],$$
  where β∈[0,1] is the discounting factor, $p_{t}=\mu \left(o_{t}\right)$ indicates the action under the state $o_{t}$, which is derived through policy μ. Thus, our objective in this paper becomes finding the optimal policy to minimize J(μ).

### 4.2. Optimizing Power Allocation Based on DDPG

**Figure 2:**
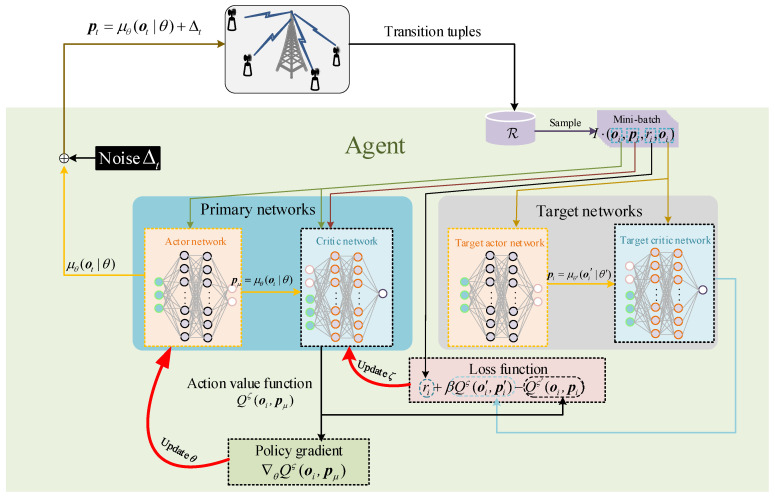
Flow diagram of DDPG.

In this subsection, we will introduce the architecture of the DDPG algorithm including primary networks (an actor network and a critic network) and target networks (a target actor network and a target critic network) [38], wherein the actor network is adopted for policy approximation and improvement, the critic network is adopted for policy evaluation and the target networks are adopted to improve the stability of the algorithm. Both primary and target networks are neural networks (DNNs). The flow diagram is shown in Figure 2. Denote θ, ζ, $\theta^{\prime }$ and $\zeta^{\prime }$ as parameters of the actor network, critic network, target actor network and target critic network, respectively, $\mu_{\theta }$ as the policy approximated by actor network and $\Delta_{t}$ as the noise added upon action for the exploration in slot *t*. Next, we will present the training stage of the DDPG algorithm in detail.

The parameters θ and ζ are first initialized randomly, $\theta^{\prime }$ and $\zeta^{\prime }$ are set as θ and ζ, respectively. In addition, a replay experience buffer R is built to cache the state transitions (lines 1–3).

Next, the algorithm loops for *E* episodes. At the beginning of each episode, the simulation parameters of the system model are reset as $u_{m,0}=0$, $p_{m,0}=1$ and $\Phi_{m,0}=0$ for each device *m*, $h_{m}\left(0\right)$ is initialized randomly. Given $p_{m,0}$ and $h_{m}\left(0\right)$, the SINR $\gamma_{m,0}$ is calculated according to Equations (2)–(4); then, the state of each device *m*, i.e., $o_{m,1}=\left[u_{m,0},\gamma_{m,0},\Phi_{m,0}\right]$ is observed by the agent (lines 4–6).

Afterwards, the algorithm iterates from slot 1 to *T*. For slot *t*, the actor network yields the output $\mu_{\theta }\left(o_{t}|\theta \right)$ under the observed state $o_{t}$ and policy μ with parameters θ. Then, a noise $\Delta_{t}$ is generated and the agent calculates the transmission powers of all devices according to $p_{t}=\mu_{\theta }\left(o_{t}|\theta \right)+\Delta_{t}$. After that, the agent calculates the $u_{m,t}$, $s_{m,t}$ and $\gamma_{m,t}$ of each device *m* according to Equations (4), (7) and (19), respectively. Afterwards, the agent calculates $\Phi_{m,t}$ and $\varepsilon_{m,t}$ according to Equations (8) and (11), respectively, and thus obtains the state of slot *t*, i.e., $o_{t+1}$, and then calculates $r_{t}$ according to Equation (21). The above tuple $\left[o_{t},p_{t},r_{t},o_{t+1}\right]$ in the replay buffer. Then, the agent inputs $o_{t+1}$ into the actor network and starts the next iteration if the number of samples in the replay buffer is not larger than *I* (lines 7–10).

If the number of tuples in the replay buffer exceeds *I*, the parameters θ, $\theta^{\prime }$, ζ and $\zeta^{\prime }$ will be updated to maximize $J(\mu_{\theta })$. Here, θ is updated toward the direction of the gradient $\nabla_{\theta }J\left(\mu_{\theta }\right)$. Specifically, the agent uniformly retrieves a mini-batch consisting of *I* tuples from the replay buffer. For each tuple *i*, i.e., $\left(o_{i},p_{i},r_{i},o_{i}^{\prime }\right)$(i∈{1,2,⋯,I}), the agent inputs $o_{i}^{\prime }$ into the target actor network and outputs $p_{i}^{\prime }=\mu_{\theta^{\prime }}\left(o_{i}^{\prime }|\theta^{\prime }\right)$, inputs $o_{i}^{\prime }$ and $p_{i}^{\prime }$ into the target critic network and outputs $Q^{\zeta^{\prime }}\left(o_{i}^{\prime },p_{i}^{\prime }\right)$ and then calculates the target value as

(23) $$y_{i}=r_{i}+\beta Q^{\zeta^{\prime }}\left(o_{i}^{\prime },p_{i}^{\prime }\right){|}_{p_{i}^{\prime }=\mu_{\theta^{\prime }}\left(o_{i}^{\prime }|\theta^{\prime }\right)}.$$

While $o_{i}$ and $p_{i}$ are the input and $Q^{\zeta }\left(o_{i},p_{i}\right)$ is the output of the critic network, the loss function can be expressed as

(24) $$L\left(\zeta \right)=\frac{1}{I}\sum_{i=1}^{I}{\left[y_{i}-Q^{\zeta }\left(o_{i},p_{i}\right)\right]}^{2}.$$

Then, the critic network is updated by the gradient descending method with the gradient of loss function $\nabla_{\zeta }L\left(\zeta \right)$ [39] (lines 11–13), i.e.,

(25) $$\zeta \leftarrow \zeta -\alpha_{c}\nabla_{\zeta }L\left(\zeta \right),$$

where $\alpha_{c}$ is the learning rate of the critic network.

After that, the agent calculates the gradient $\nabla_{\theta }J\left(\mu_{\theta }\right)$ as [40]

(26) $$\begin{array}{cc} \; & \nabla_{\theta }J\left(\mu_{\theta }\right)\\ \; & \approx \frac{1}{I}\sum_{i=1}^{I}\nabla_{\theta }Q^{\zeta }\left(o_{i},p_{\mu }\right){|}_{p_{\mu }=\mu_{\theta }\left(o_{i}|\theta \right)}\\ \; & =\frac{1}{I}\sum_{i=1}^{I}\nabla_{\theta }\mu_{\theta }\left(o_{i}|\theta \right)\cdot \nabla_{p_{\mu }}Q^{\zeta }\left(o_{i},p_{\mu }\right){|}_{p_{\mu }=\mu_{\theta }\left(o_{i}|\theta \right)} \end{array},$$

where the chain rule is applied to derive the gradient of $Q^{\zeta }\left(o_{i},p_{\mu }\right)$ with respect to θ [40]. Given $\nabla_{\theta }J\left(\mu_{\theta }\right)$, the actor network can be updated by gradient ascending to maximize $J(\mu_{\theta })$, i.e.,

(27) $$\theta \leftarrow \theta +\alpha_{a}\nabla_{\theta }J\left(\mu_{\theta }\right),$$

where $\alpha_{a}$ is the learning rate of the actor network.

After the parameters of the primary networks are updated, the parameters of the target networks are updated based on the parameters of primary networks, i.e.,

(28) $$\begin{array}{cc} \zeta^{\prime } & \leftarrow \kappa \zeta +\left(1-\kappa \right)\zeta^{\prime }\\ \theta^{\prime } & \leftarrow \kappa \theta +\left(1-\kappa \right)\theta^{\prime } \end{array}\;,$$

where κ is a constant much smaller than 1, i.e., κ≪1 (line 15).

Up to now, the iteration for slot *t* is finished and the agent starts the next iteration until the number of slots reaches *T*. Then, the agent starts the next episode. When the number of episodes reaches *E*, the training stage is finished and outputs the near-optimal policy. The pseudo-code of the training stage is described in Algorithm 1.

Next, the testing stage is initialized to test the performance under the near-optimal policy. Compared with the training stage, the parameter-updating process is omitted in the testing process and actions in each slot are generated by the near-optimal policy. The corresponding pseudo-code is shown in Algorithm 2, where $\theta^{*}$ is the parameter to achieve the near-optimal policy in the training stage.
**Algorithm 1:** Training stage of the DDPG algorithm
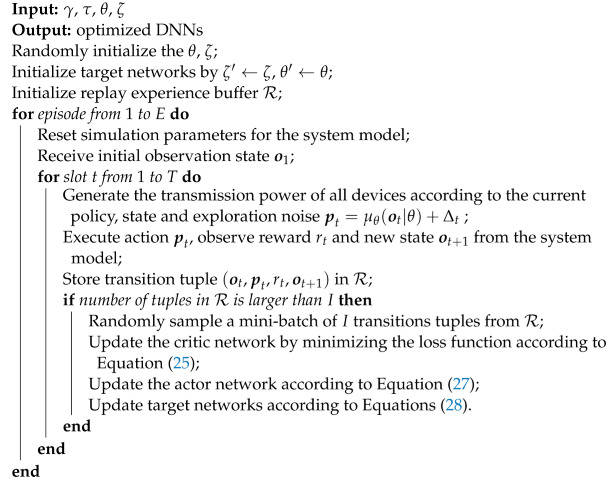


**Algorithm 2:** Testing stage of the DDPG algorithm

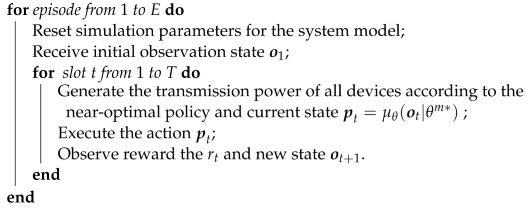



### 4.3. Complexity Investigation

In this subsection, we investigate the complexity of the proposed algorithm. Denote $G_{P}$ and $U_{p}$ as the computational complexity for computing gradients and updating parameters of the primary networks, respectively. Since the architecture of the target networks is the same as that for the primary networks, the computational complexity for updating the parameters of the target networks is also the same as the one for the primary networks. The complexity of the proposed algorithm is related to the number of slots in the training process. To be specific, during each slot, the primary networks calculate the gradients and updating parameters, while the target networks update the parameters with the parameters of the primary networks according to Equation (28). Moreover, we denote the complexity of calculating sample decisions based on power allocation as $S_{d}$. Thus, the complexity of the proposed algorithm in a slot is $O(G_{P}+2U_{P}+S_{d})$. Note that the gradients calculation and parameters updating will be processed until the number of tuples cached in the replay buffer exceeds *I*. The proposed algorithm will loop for *E* episodes, each of which contains *T* slots. Thus, the complexity of the proposed algorithm can be expressed as $O(\left(E\cdot T-I\right)\left(G_{P}+2U_{P}+S_{d}\right))$.

## 5. Simulation Results and Analysis

In this section, we provide simulation results to verify the effectiveness of the proposed power allocation strategy. The scenario is described in the system model. The experiments were conducted during the training and testing phases. The simulation tool was Python 3.6. In the simulation, both the actor network and critic network are the four-layer fully connected DNN with two hidden layers which are equipped with 400 and 300 neurons, respectively. The Adam optimization method [41] is adopted to update the parameters of the critic network and actor network. The noise $\Delta_{t}$ (for exploration) follows the Ornstein–Uhlenbeck (OU) process with decay-rate 0.15 and variation 0.004, respectively [42]. The small-scale fading of each device is initialized by white Gaussian noise, and the Rayleigh block fading model is employed to simulate the stochastic small-scale fading [43]. The reference channel gain of each device is −30 dB when the communications distance is 1 m, the path-loss exponent is 2 and the communication distances is randomly set within a range of [50,100] meters. The parameters of the measurement setup and DDPG algorithm are set according to [13] and [39], respectively, which are shown in Table 3.

### 5.1. Training Stage

**Figure 3:**
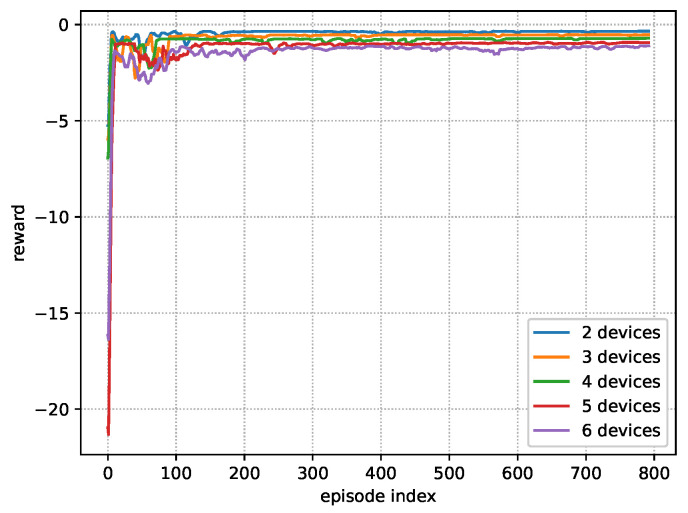
Learning curves under various number of devices.

Figure 3 shows the learning curves in the training stage, i.e., rewards in different episodes, for different numbers of IoT devices. It can be seen that the rewards of different curves rise and fluctuate from episode 0 to 150, which reflects that the agent is learning the policy to maximize the average reward. After that, the learning curves turn out to be stable, which indicates that the near-optimal policy has been learned by the agent. Note that there is a litter jitters after episode 150, which is due to the fact that the agent is adjusting slightly since the exploration noise prevents the agent from converging into the local optima. It can also be seen that the large number of devices incurs a low reward. This is attributed to the fact that each device will be affected by more interference as the number of devices in the system increases, which leads to the lower transmission rate. This will prolong the transmission delay and further increase the AoI of the system. Then, the BS may inform the devices to consume more energy to sample more frequently and transmit faster; thus, the lower AoI can be guaranteed.

### 5.2. Testing Stage

In the testing stage, we verify the performance of the near-optimal policy obtained in the training stage. Existing works have adopted GA [32] and random power allocation policy [33] as the baseline algorithm for power allocation; therefore, we selected these two algorithms for comparison. Here, random power allocation policy and GA are introduced as follows:

- Random policy: Randomly allocate the power of each device *m* within $\left[0,P_{m,max}\right]$ and the sample collection commands is obtained according to Equation (19).
- GA-based power allocation: In each time slot, the BS randomly generates a population vector according to $P_{m,max}$ and a population size *B*. Each individual element in the population vector stands for the power allocation for all devices. The BS selects the best individuals in the population vector as offspring according to their fitness, i.e., the reward function, of each individual. Then, after evolving for $N_{GA}$ times, for each evolution, the probabilities of crossover and mutation for these offspring become $F_{c}$ and $F_{m}$, respectively, where crossover means that two individuals in the offspring exchange the power allocation of a random device, and mutation means that the power allocation of any device in the offspring is selected within $\left[0,P_{m,max}\right]$ randomly. After that, selecting best individuals from the offspring that has experienced crossover and mutation as the input for the next evolution. After all the evolutions, the best individual from the last offspring, which is the near-optimal power allocation derived by GA, is elected. After that, the BS calculates the optimal sample collection based on the near-optimal power allocation derived by GA according to Equation (19), and then executes the near-optimal power allocation derived by GA and the optimal sample collection. In the end, the BS iterates into the next time slot.

**Figure 4:**
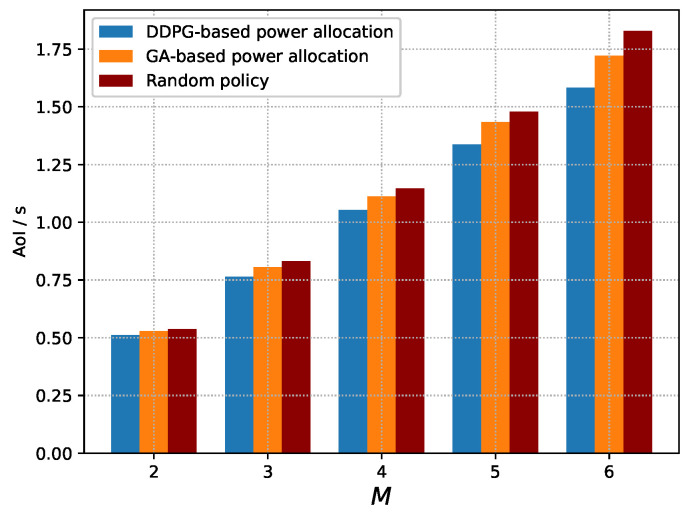
AoI of the system vs. number of devices.

Figure 4 presents the AoI of the near-optimal policy derived by DDPG, the random policy and the near-optimal power allocation derived by GA. It can be seen that the AoI of the three policies increases as the number of devices increases. This is because each device will suffer from the interference as the number of devices increases, and thus degrades its transmission delay according to Equation (9), which may further increase the AoI of the system. Meanwhile, the near-optimal policy derived by DDPG and the near-optimal power allocation derived by GA always outperform the random policy, because the near-optimal policy derived by DDPG can adjust the power allocation adaptively according to the observed state, and the near-optimal power allocation derived by GA will find the optimal power allocation according to the fitness in the evolutions to ensure a low AoI, while the random policy just generates power allocation randomly. It also can be seen that the near-optimal policy derived by DDPG outperforms the near-optimal power allocation derived by GA, because the DDPG will consider the influence of the power allocation in each time slot on the subsequent AoI, while GA cannot.

**Figure 5:**
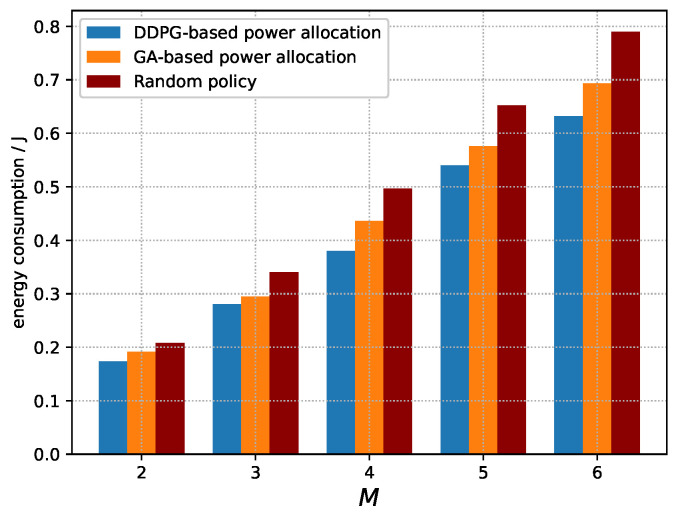
Energy consumption of the system vs. number of devices.

Figure 5 compares the energy consumption of three policies. It can be seen that energy consumption increases as the number of devices increases. This is due to the fact that, according to Equation (4), the increasing number of vehicles increases the interference power, leading to a decrease in SINR. According to Equation (9), the AoI of the system increases as the SINR decreases. Hence, the devices may consume more energy for more frequent sampling and faster transmission to reduce AoI. Moreover, the increasing number of devices contributes to the increasing energy consumption according to Equation (12). Meanwhile, the near-optimal policy derived by DDPG and the near-optimal power allocation derived by GA always outperform the random policy, because DDPG and GA can allocate power adaptively to ensure a low-energy consumption. Moreover, it also can be seen that the near-optimal policy derived by DDPG always outperforms the near-optimal power allocation derived by GA, which is due to the fact that the GA cannot take into account the influence of the power allocation in each time slot on the subsequent energy consumption.

**Figure 6:**
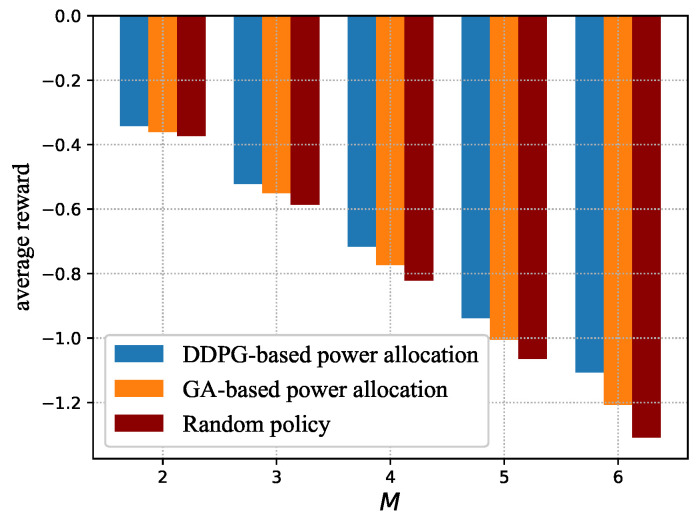
Average reward vs. number of devices.

Figure 6 compares the average reward under the three policies, where the reward is obtained by averaging the test results over all slots. We can see that the average reward decreases as the number of devices increases. This is due to the fact that the reward function consists of the AoI and energy consumption of the system according to Equation (21), and both of them increase as the number of devices increases. Moreover, the average reward under the near-optimal policy derived by DDPG and the near-optimal power allocation derived by GA are higher than that of the random policy. This is attributed to the fact that the near-optimal policy allocates power according to the observed state to maximize the long-term discounted reward, and the GA obtains the near-optimal power allocation by maximizing the reward. It can also be seen that the near-optimal policy obtained by the DDPG-based method always outperforms the near-optimal power allocation derived by the GA. This is due to the fact that the GA aims to find the near-optimal power allocation based on fitness, i.e., the reward in each slot, while ignoring the long-term reward maximization.

**Figure 7:**
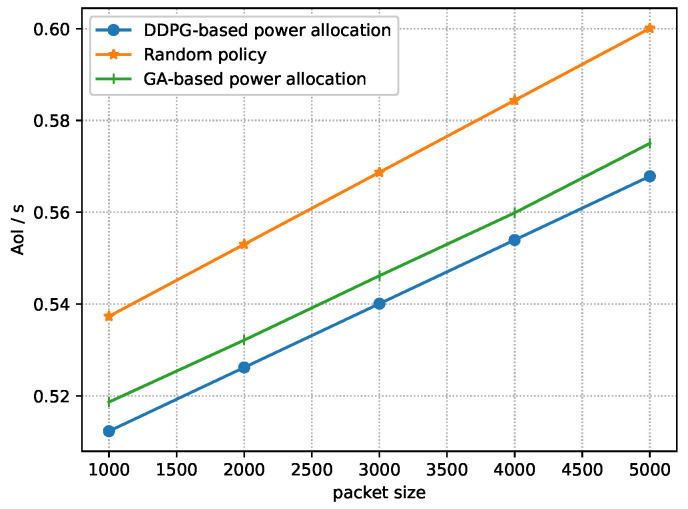
AoI of the system vs. packet size.

Figure 7 shows the relationship between the AoI of the system and packet size, i.e., *Q*, under three policies. It can be seen that the AoI increases as the packet size increases under the three policies. This is due to the fact that, according to Equation (9), the packet size influences the transmission delay. That is, the transmission delay is long when the packet size is large. With regards to Equation (8), the AoI is affected by transmission delay, wherein a smaller transmission delay results in a smaller AoI. In addition, we can see that the AoI of the near-optimal policy obtained by DDPG and the near-optimal power allocation derived by the GA are lower than the AoI under the random policy. This is because the near-optimal policy derived by DDPG can adjust the power allocation based on the observed state, and the GA obtain near-optimal power allocation according to fitness, which can significantly reduce the AoI of the system. The gap between the near-optimal power allocation derived by DDPG and the near-optimal power allocation derived by the GA is caused by the advantage of long-term minimization for DDPG.

**Figure 8:**
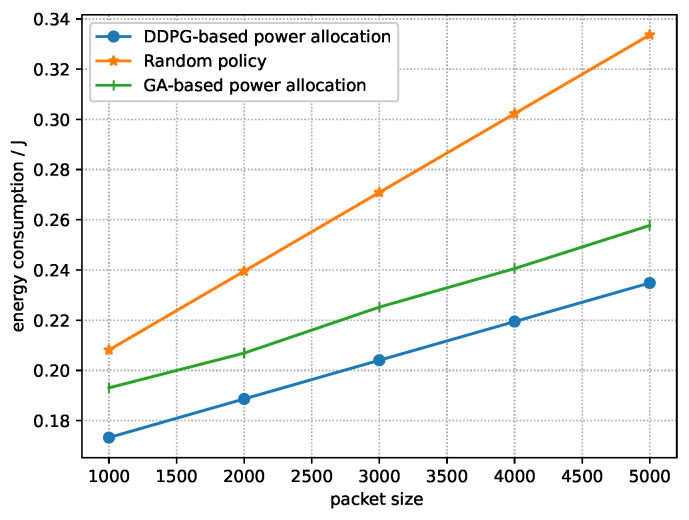
Energy consumption vs. packet size.

Figure 8 shows the relationship between the energy consumption of the system and packet size under three policies. It can be seen that the energy consumption of all three policies increases when the packet size increases. As shown in Figure 7, the transmission delay is long when the packet size is large, thus incurring the increase in energy consumption of the system. We can also see that the energy consumption of the near-optimal policy derived by DDPG and the near-optimal power allocation derived by the GA are lower than that of the random policy. This is due to the fact that the near-optimal policy derived by DDPG can adaptively allocate power and the GA can obtain the near-optimal power allocation according to fitness to ensure a lower energy consumption. However, the near-optimal policy derived by DDPG accounts for the influence of power allocation on the energy consumption of later time slots; thus, the near-optimal policy obtained by DDPG has a lower energy consumption than the near-optimal power allocation derived by the GA.

## 6. Conclusions

In this paper, we formulated a problem to minimize the AoI and energy consumption of the MIMO-NOMA IoT system. To solve it, we simplified the formulated problem and proposed the power allocation scheme based on DDPG to maximize the long-term discounted reward. Extensive simulations have demonstrated that the proposed scheme reduces the reward by 6.44% compared to the GA, and by 11.78% compared to the random policy, respectively. According to the theoretical analysis and simulation results, the key findings and contributions of this paper can be summarized as follows: (1) An increase in the number of devices and packet size will increase the AoI of the system. In this case, agents can inform the devices to consume more energy to sample more frequently and transmit faster, thereby reducing the AoI and increasing the energy consumption. (2) The near-optimal policy trained by DDPG outperforms the baseline policy under different numbers of users and packet sizes, which has a good capability to suit the system dynamic variation. We also noted some limitations and future directions for further research in this study: DDPG may face challenges when addressing high-dimensional state and action spaces. In future work, we will consider decomposing the problem into multiple subtasks for independent learning or improving the function approximators to enhance its robustness. In addition, as mentioned in [44], fairness is also a relatively important factor in the NOMA system. Therefore, our future research will focus on achieving a fair resource allocation in MIMO-NOMA systems and evaluating its impact on other performances.

## Figures and Tables

**Table 1:** The comparison between related works.

Related Work	MIMO-NOMA	AoI Minimization	Energy Optimization
[12,15,17,18,19]	×	✓	×
[13,14,16,22]	×	✓	✓
[25,28,29,32,33]	✓	×	✓
[23,24,26,27]	✓	×	×

**Table 2:** The summary of the notations.

Notation	Description	Notation	Description
*B*	Population size of genetic algorithm.	$C_{s}$	The energy consumption to sample fresh information and generate upload packet.
$c_{m,t}$	Complex data symbol with 1 as variance.	$d_{m}$	The communication distance between device *m* and BS.
*E*	Number of episodes.	$F_{c}/F_{m}$	Probability of offspring in genetic algorithm for crossover/mutation.
$G_{P}/U_{P}$	Complexity of the primary networks for computing gradients/updating parameters.	$h_{m}\left(t\right)$	The channel vector between device *m* and BS in slot *t*.
*i*	Index of transition tuples in mini-batch.	*I*	The number of transition tuples in a mini-batch.
$I_{m}$	The set of devices in which the received power is weaker than device *m*.	J(μ)	The long-term discounted reward under policy μ.
*K*	The number of antennas equipped in BS.	$l_{m,t}$	The transmission delay of device *m* in slot *t*.
*L*	Loss function.	n(t)	Additive white Gaussian noise.
$N_{GA}$	Evolution times of genetic algorithm	m/M/M	Index/number/set of devices.
$o_{t}/o_{m,t}$	State in slot *t* of all devices/device *m*	$p_{t}$/$p_{m,t}$	Transmission power of all devices/device *m*.
$P_{m,t}$	Maximum transmission power device *m*.	$Q(o_{t},p_{t})$	Action-value function under $o_{t}$ and $p_{t}$.
$Q(o_{t},p_{t})$	Action-value function under $o_{t}$ and $p_{t}$.	*Q*	Packet size.
$r_{t}$	Reward function.	$s_{t}$/$s_{m,t}$	Indicator of sample or not for all devices/device *m*.
$s_{t}$/$s_{m,t}$	Indicator of sample or not for all devices/device *m*.	$S_{d}$	Complexity of calculating sample decisions based on power allocation.
$S_{d}$	Complexity of calculating sample decisions based on power allocation.	*t*/T	Index/set of slot.
U	The set of undecoded received power of BS.	$u_{t}$/$u_{m,t}$	Indicator of transmission success for all devices/device *m*.
*W*	Bandwidth of system.	$\alpha_{a}$/$\alpha_{c}$	Learning rate of actor network/critic network.
β	Discounting factor.	$\gamma_{a}$, $\gamma_{c}$	Weighted factors of reward function.
$\Gamma_{m,t}$	Received power of BS for device *m* in slot *t*.	$\Delta_{t}$	Exploration noise.
$\varepsilon_{m,t}$	The energy consumed by device *m* in slot *t*.	$\bar{\varepsilon }$	The average sum energy consumption in slot *t*.
ζ/$\zeta^{\prime }$	Parameters of critic-network/target critic-network.	θ/$\theta^{\prime }$/$\theta^{*}$	Parameters of actor-network/target actor-network/optimal policy.
κ	The constant for the update of target networks.	$\mu_{\theta }$	Policy approximated by actor-network with θ.
$\pi_{m,t}$	Transmission rate of device *m* in slot *t*.	$\rho_{m}$	Normalized channel correlation coefficient.
$\sigma_{R}^{2}$	Variance of received signal’s noise.	$\phi_{m,t}$/$\Phi_{m,t}$	AoI of device *m* in slot *t* on device/BS.
$\bar{\Phi }$	The average sum AoI.		

**Table 3:** Values of the parameters in the experiments.

Parameters of System Model [13]			
**Parameter**	**Value**	**Parameter**	**Value**
τ	0.1 s	*K*	4
*W*	18 kHz	$C_{s}$	0.5 J
$P_{m,max}$	2 W	*T*	500
Parameters of Agent [39]			
**Parameter**	**Value**	**Parameter**	**Value**
κ	0.001	*I*	64
*E*	800	β	0.99
|R|	$2.5\times 10^{5}$	$\gamma_{e}$	0.5
$\gamma_{a}$	0.5	$\alpha_{a}$	$10^{-3}$
$\alpha_{c}$	$10^{-4}$	$F_{c}/F_{m}$	0.8/0.5
*B*	10	$N_{GA}$	50

## Data Availability

Data are contained within the article.
